# Correlation of Antiglobulin Reactivity and Severity of Pancytopenia in a Patient with Hemophagocytic Lymphohistiocytosis: A Case Report and Review of Literature

**DOI:** 10.7759/cureus.711

**Published:** 2016-07-26

**Authors:** Uroosa Ibrahim, Muhammad N Siddique, Gautam Valecha, Masoud Asgari, Edhan Isaac, Meekoo Dhar

**Affiliations:** 1 Hematology/Oncology, Staten Island University Hospital; 2 Medicine, Staten Island University Hospital; 3 Pathology, Staten Island University Hospital; 4 Transfusion Medicine, Staten Island University Hospital

**Keywords:** hemophagocytic lymphohistiocytosis, autoimmune hemolytic anemia, aggressive, rare

## Abstract

A 46‑year‑old obese male with a medical history of thalassemia minor presented to the emergency room with complaints of severe fatigue and jaundice worsening over two weeks. On further evaluation, the patient was found to have significant hyperbilirubinemia and transaminitis. The hospital course was further complicated by pancytopenia requiring multiple transfusions, worsening hyperbilirubinemia, severe hyperferritinemia, hypofibrinogenemia, and hypertriglyceridemia. He was also found to have splenomegaly and evidence of hemophagocytosis on bone marrow biopsy. On further testing, the patient was also found to have evidence of hemolysis along with a positive direct Coomb's test consistent with autoimmune hemolytic anemia (AIHA), and elevated soluble IL-2 receptor level. The patient was subsequently diagnosed with hemophagocytic lymphohistiocytosis (HLH). He was treated with HLH-94 protocol along with rituximab for AIHA which resulted in improvement of patient's condition. We present a case of HLH with no prior history of autoimmune disease, associated with Coomb's positive AIHA that resolved after therapy for HLH. Our case also delineates how the intensity of antiglobulin reactivity, if present, may correlate with severity of the disease, its progression, and response to treatment.

## Introduction

Hemophagocytic lymphohistiocytosis is a rare hematologic disorder resulting from the disruption of immune homeostasis. It is an aggressive and life-threatening condition characterized by a severe inflammatory state driven by excessive cytokine production. If not treated promptly, HLH can be rapidly fatal. However, due to the rarity of this condition and variable clinical presentation, the diagnosis is often delayed, resulting in poor outcomes. HLH can occur as a sporadic or familial disorder. It can be triggered by a variety of events that disrupt immune regulation. Commonly, infection is one such trigger. A possible role of autoimmune hemolysis in erythrocytopenia encountered in HLH is not formally described in the literature. We present a case of HLH with antiglobulin reactivity correlating with the disease activity and severity of pancytopenia.

## Case presentation

A 46‑year‑old obese male with a medical history of thalassemia minor presented to the emergency room with complaints of severe fatigue and jaundice worsening over two weeks. He denied having any abdominal pain, change in bowel habits, melena, or rectal bleeding. He did not have any of the following: fever, chills, weight loss or night sweats. There was no history of recent travel, toxic exposure, blood transfusions or sick contacts. He was not on any medications or herbal preparations and denied smoking, heavy alcohol use or illicit drug use. His past surgical history included a remote laparoscopic cholecystectomy and Roux-En-Y gastric bypass surgery. The patient worked as an emergency medical services personnel, and his family history was significant for Cooley's anemia. On physical examination, the patient appeared fatigued. Skin and sclerae were significantly icteric. No mucocutaneous lesions were noted. No lymphadenopathy was appreciated. Abdomen was soft with no palpable organomegaly. He was afebrile and hemodynamically stable. The rest of the physical examination was unremarkable.

On admission, laboratory studies revealed a total bilirubin of 15.9 mg/dL; direct bilirubin being 1.6 mg/dL. Serum alkaline phosphatase level was within the normal range. Serum alanine aminotransferase (ALT) and aspartate aminotransferase (AST) were both slightly elevated at 63 mg/dL and 44 mg/dL, respectively. Hemoglobin (Hb) was 10.6 g/dL with a mean corpuscular volume (MCV) of 65.5 fL, white blood cell (WBC) count of 7.8 TH/mm^3^ and platelet count of 309 TH/mm^3^. Coagulation studies were normal. Blood Urea Nitrogen (BUN) was 27 mg/dL, and serum creatinine was 0.61 mg/dL. Serum iron was 319 ug/dL, total iron binding capacity (TIBC) was 319 ug/dL, percentage saturation was 100%, ferritin was 1267 ng/ml, folate was 4.6 ng/ml and vitamin B12 level was 379 pg/ml. Serum protein electrophoresis (SPEP) was negative for any monoclonal protein. Lactate dehydrogenase (LDH) was high at 545 IU/L, but, reticulocyte count was only 0.79%, and serum haptoglobin was 82 mg/dL. A direct antiglobulin test performed at our facility was negative. A blood sample was sent out to American Red Cross Blood services Southern California region for confirmation. We later learned that when the test was done with standard tube technique, it was indeed negative for anti-IgG, but turned out to be positive with Cold LISS wash technique.

A computed tomography (CT) scan of the abdomen revealed an enlarged spleen measuring 20 cm. Notably, the scan did not show any evidence of retroperitoneal hemorrhage, any intrahepatic or extrahepatic biliary duct dilatation, or any abdominal lymphadenopathy. Thoracic lymphadenopathy or any other lung pathology was also ruled out with a CT scan of the chest.   

While the diagnostic workup was being done, the patient’s anemia worsened. He was poorly responsive to transfusions and required multiple transfusions daily. His Hb dropped down to 4.1 g/dL at one point. Other blood counts showed a decline too. The lowest WBC count was 3.48 TH/mm^3^, and platelet count was 80 TH/mm^3^. Total bilirubin started rising. At one point, it reached 71.9 mg/dL. Alkaline phosphatase remained normal, and a repeat abdominal CT scan ruled out any surgical cause of jaundice. Plasmapheresis was initiated, and the total bilirubin levels decreased to 23.8 mg/dL. Although the reticulocyte count hovered between 0.5 to 1% during this time, serum haptoglobin decreased to less than 3 mg/dL, and serum LDH rose up to 1558 IU/L. At this point, a repeat direct antiglobulin test was sent. We later learned that the test was weakly positive with standard tube technique, but strongly positive with Cold LISS wash technique.

A repeat ferritin showed a significant rise to 16,693 mg/dL. Extensive workup ensued to explain the hyperferritinemia, extreme hyperbilirubinemia, and pancytopenia. Viral hepatitis panel, anti-smooth muscle antibody (SMA) and anti-mitochondrial antibody were negative. A liver biopsy revealed cholestasis and suspected hemochromatosis. HFE gene mutation analysis revealed a heterozygous H63D mutation and was negative for the C282Y mutation. Epstein-Barr virus (EBV) serology as well as DNA PCR, parvovirus IgM, IgG antibodies, and HIV expedited assay were all negative. Anti-cytomegalovirus (CMV) IgG antibody was positive, but the PCR study was negative. A peripheral blood flow cytometry ruled out PNH clone. Bone marrow aspiration and biopsy revealed normocellular bone marrow with trilineage hematopoiesis and erythroid hyperplasia without excessive blasts. Flow cytometry was negative for any evidence of lymphoma or leukemia. Notably, the pathologist noted a few foci of erythrophagocytosis. A bone marrow biopsy was repeated a week later, and it confirmed the same findings.

At this point, a diagnosis of the hemophagocytic syndrome was contemplated. The suspicion strengthened when serum triglyceride was found to be elevated at 338 mg/dL. Fibrinogen level was 671 mg/dL. At this point, a blood sample for soluble IL-2 receptor level was sent. However, the patient had already fulfilled the minimal requirement of meeting five out of the eight diagnostic criteria for HLH (see Table 1). As we could not identify any triggering condition and the patient was acutely ill, we decided to treat the patient with the HLH-94 protocol. CNS involvement was ruled out by an MRI of the brain and two lumbar punctures. 

**Table 1:** Hemophagocytic lymphohistiocytosis diagnostic criteria

Diagnostic Criteria for HLH Used in HLH - 2004 Trial
1. Molecular diagnosis consistent with HLH (e.g. pathologic mutations of PRF1, UNC13D, STX11, SH2D1A, LYST, ITK, SLC7A7, XMEN, HPS or BIRC4)
OR
2. Five of the following eight criteria:
I). Fever ≥ 38.5⁰C
II). Splenomegaly
III). Cytopenias (affecting at least 2 of 3 lineages in peripheral blood)
a). Hb < 9 g/dL (in infants < 4 weeks: Hb < 10 g/dL)
b). Platelets < 100 x 10^3^/mL
c). Neutrophils < 1 x 10^3^/mL
IV). Hypertriglyceridemia (fasting > 265 mg/dL) and/or hypofibrinogenemia (< 150 mg/dL)
V). Hemophagocytosis in bone marrow, spleen, lymph nodes or liver
VI). Low or absent NK cell activity
VII). Ferritin > 500 ng/mL
VIII). Elevated soluble CD25 (that is, soluble IL-2 receptor alpha) 2 standard deviations above age-adjusted laboratory specific norms

Subsequently, the results of the first Coombs test sent out to California were reported positive. Thus, a component of his anemia and hyperbilirubinemia could now be attributed to AIHA. We proceeded concurrently with rituximab and HLH‑94 protocol. Altogether, the patient received four doses of rituximab. Dexamethasone was started at 10 mg/m^2^, and etoposide at 75 mg/m^2^ with a 50% reduction in view of marked hyperbilirubinemia. The patient was monitored in intensive care unit (ICU).

As per the protocol, the patient should have received etoposide twice a week for the first two weeks of treatment, but due to chemotherapy-related cytopenias, he ended up receiving chemotherapy only once a week. During this period, the patient was maintained on supportive care with transfusions as needed.

The patient’s soluble IL-2 receptor was elevated at 1517 U/mL. We also sent his peripheral blood sample to a reference laboratory in Cincinnati Children's Hospital, Ohio for further specialized studies. X-linked Inhibitor of Apoptosis (XIAP) level and Natural killer (NK) cell function were both decreased. XIAP levels in CD4, CD8 and CD56 cells were: 75% (normal >= 92%), 78% (normal >= 93%) and 78% (normal >= 94%), respectively. NK Cell function was 54% (normal range 47 - 95%). We later found that he did not have any mutations with known association to the hemophagocytic syndrome, such as STX11, Munc13-4, STXBP2, RAB27A, or PRF1 mutation. SH2D1A mutation and BIRC4 mutation associated with X-linked lymphoproliferative diseases were also negative.

A total of six doses of etoposide were administered during the hospital stay which progressively led to decreased transfusion requirements and eventually rendered him transfusion independent, with Hb stabilized at around 9 g/dL and other counts normalized. His aminotransferase levels were improved. Total bilirubin and LDH improved, and stabilized to less than 3 mg/dL and 300 IU/L, respectively.  His ferritin levels had started to show a gradual decline and reached 4666 mg/dL at this point. A repeat direct antiglobulin test for IgG turned negative with the standard tube method as well as with Cold LISS wash technique.

The patient was considered stable for discharge. He was to complete a total of ten doses of etoposide along with dexamethasone that was gradually tapered over about six weeks after the last dose of etoposide. During this time he was on antimicrobial prophylaxis with fluconazole, valacyclovir, and co-trimoxazole that was continued for three months after completing his treatment with etoposide. He tolerated the treatment fairly well apart from steroid-induced hyperglycemia which was managed with insulin. A post-treatment serum soluble IL2 receptor, fibrinogen, and serum triglyceride showed complete normalization. His Hb gradually improved further and returned to his baseline about three months after receiving etoposide. At that time, it was 12.2 g/dL along with normal WBC and platelet counts.

The patient’s ferritin, however, remained elevated and ranged between 4500 mg/dL to 5000 mg/dL until about six months after finishing his chemotherapy when it almost halved to 2548 mg/dL. This persistent elevation was at least in part attributed to metabolic dysfunction such as him being obese along with steroid-induced hyperglycemia. Indeed, with better glycemic control, it showed a further decline and the latest ferritin was 1746 mg/dL. 

Consent was waived.

## Discussion

HLH is a rare hematologic disorder resulting from excessive immune activation. It is an aggressive condition that results from unchecked proliferation and activation of functionally benign lymphocytes and macrophages due to the dysregulation of their function by poorly functioning cytotoxic lymphocytes and NK cells [1]. The resultant cytokine storm and activated macrophages are responsible for most of the clinical manifestations of this disease including hypertriglyceridemia, hypofibrinogenemia, decreased hematopoiesis, hemophagocytosis and multiorgan failure [2].

If not rapidly treated, HLH can result in significant mortality and morbidity. However, the diagnosis is often delayed because of its variable presentation and rarity. It appears that fewer cases are missed now, at least at the tertiary pediatric centers. Significantly higher when compared to a series from the 1970s that reported an incidence of 1.2 children per million per year, a recent review of HLH cases from large pediatric hospitals in Texas, United States revealed a calculated cross-sectional prevalence of one in 100,000 children [3].

This disorder can be both familial and sporadic. The familial form primarily affects the pediatric population. These patients are genetically predisposed due to well-known mutations such as STX11, Munc13‑4, STXBP2, RAB27A, PRF1, LYST and AP3 mutations. On the other hand, most of the adult cases are sporadic and are triggered by the infectious, inflammatory or malignant process. Therefore, diagnosis in the adults is based on a constellation of clinical criteria (Table 1) [4].

One of the most important clinical issues in HLH is anemia, whose mechanism has not been well-elucidated. It has been postulated to be mainly due to the increased destruction of RBC's via hemophagocytosis by the overactive macrophages [5]. However, as many patients with HLH show decreased bone marrow cellularity, bone marrow failure with reduced red cell production has also been hypothesized, and indeed was mechanistically described recently. CD47 is a transmembrane protein located on hematopoietic stem cells that work in collaboration with signal-regulatory protein alpha (SIRPA) to prevent phagocytosis. Its expression can be down-regulated by hypercytokinemia in HLH resulting in increased hemophagocytosis [6]. Besides this, in vivo murine models have clearly demonstrated the role of IFN-γ in macrophage activation that leads to hemophagocytosis [7].

A possible role of autoimmune hemolysis in the pathogenesis of erythrocytopenia encountered in HLH is not formally described in the literature. It has, nevertheless, been seen in settings where HLH occurs in association with an autoimmune disease, and in association with pregnancy [8-9]. Whether its presence in our patient suggests an independent role of AIHA in the mechanism of anemia in certain cases of HLH, or merely exemplifies another manifestation of immune dysregulation seen in HLH, or represents a red flag that warns about the possibility of an imminently developing immunodysregulatory process such as HLH, is obviously difficult to differentiate. The possibility that it merely reflects an incidental concurrent process is unlikely because of the strong correlation observed between the intensity of direct antiglobulin reactivity and disease severity. In our report, not only was the direct antiglobulin test positive before the full spectrum of clinical characteristics appeared, but also the strength of the test positivity increased as the severity of the disease progressed. This observation strongly suggests the possibility that AIHA represented an integral component in the pathophysiology of HLH in our case, which makes it unique. Additionally, this implies that indeed if such underlying pathophysiologic component is identified in a given case, the role of direct antiglobulin testing deserves consideration for disease monitoring. It is well established that assessing the responsiveness promptly is immensely important as the initiation of an alternative treatment plan is needed for patients whose HLH has not completely resolved by the eighth week. Moreover, HLH is prone to reactivation from triggers of the immune response such as new or persistent underlying infection. Disease severity in HLH is highly correlated with soluble IL-2 receptor levels and with NK-cell function assays [4]. However, these are highly specialized tests that are done in only a few reference laboratories in the United States, and the turn out time is at least two weeks. Given the rapidity with which HLH progresses, waiting for these results is not practical. The retrospective analyses show that highly elevated levels of serum ferritin (more than 10,000 ng/ml) are considered 90% sensitive, and 96% specific for the diagnosis of HLH [10]. The treatment resulting in a rapid and deep decline in ferritin levels indicates decreased mortality [11] as well. Yet, the correlation with disease severity is not perfect. Being an acute phase reactant, it may remain elevated due to a concurrent non-HLH process even in the context of an improving HLH, a phenomenon, our case clearly demonstrates.

## Conclusions

The above-described limitations of the current existing treatment monitoring modalities, on one hand, entail the need for more rigorous basic science work. Such work will allow discovery of new disease markers with rapid turnover times. On the other hand, this course will encourage the physicians to search for existing correlative clinical and basic laboratory parameters. Such correlation may provide clues to disease activity, progression, and response. We now share our clinical experience that plainly rolls out how the intensity of antiglobulin reactivity, if present, may correlate with disease severity, progression and response to treatment. 
